# Study on the Li^+^ Desolvation Mechanism of Doped Carbon Nanotubes and Their Regulatory Effect on the Performance of Organic Supercapacitors

**DOI:** 10.3390/ma19194090

**Published:** 2026-09-24

**Authors:** Yuwei Si, Fudong Liu, Bo Yang, Shuai Yu, Yinping Chen, Beiqi Zhang

**Affiliations:** School of Metallurgy and Materials Engineering, Liaoning Institute of Science and Technology, Benxi 117004, China; siyuwei2006@163.com (Y.S.); boyangustb@163.com (B.Y.); yushuai717@163.com (S.Y.); neuchenyinping@126.com (Y.C.); z2442192395@163.com (B.Z.)

**Keywords:** single-walled carbon nanotubes, N/P doping, desolvation, organic supercapacitors, first-principles calculation

## Abstract

Organic supercapacitors (OSCs) suffer from sluggish Li^+^ desolvation and ion diffusion kinetics, limiting their electrochemical performance and practical application. Heteroatom doping is a valid strategy to improve the capacitive properties of carbon electrode materials. Herein, density functional theory (DFT) first-principles calculations were adopted to systematically investigate and compare the effects of equal-concentration N and P doping on the Li^+^ desolvation behavior, capacitance performance, and ion diffusion kinetics of single-walled carbon nanotubes (SWCNTs) in acetonitrile-based electrolytes. The results show that N doping maintains the [Li(AN)]^+^ critical desolvation size (5.91 Å) of pristine SWCNTs, while P doping increases this size to 6.11 Å. Benefiting from the optimized desolvation behavior, P-doped SWCNTs achieve a maximum relative capacitance 1.9 times that of pristine SWCNTs, outperforming N-doped SWCNTs (1.3 times). Moreover, P doping significantly reduces the [Li(AN)]^+^ diffusion barrier to 0.99 eV, much lower than that of pristine and N-doped SWCNTs, thereby accelerating ion migration. Electronic structure analysis confirms that heteroatom doping weakens electron localization and C-heteroatom covalent bonding, and the weaker P-C bonding further accounts for the superior performance of P-doped SWCNTs. This study clarifies the intrinsic regulatory mechanism of N/P doping on Li^+^ desolvation and electrochemical performance of SWCNTs, providing a theoretical guideline for the design of high-performance carbon-based electrodes for OSCs.

## 1. Introduction

With the escalating global demand for high-performance energy storage devices, organic supercapacitors (OSCs) have emerged as promising candidates in fields such as electric vehicles, portable electronics, and renewable energy systems, owing to their high power density, long cycle life, and wide operating temperature range [1,2,3]. However, the relatively low energy density and limited ionic diffusion kinetics of OSCs, primarily due to sluggish Li^+^ desolvation and restricted ion migration within carbon electrodes, remain critical bottlenecks to their large-scale commercial application [4,5]. Carbon-based materials, including carbon nanotubes (CNTs), graphene, and porous carbon, are widely adopted as OSC electrode materials due to their high specific surface area, excellent electrical conductivity, and chemical stability [6,7,8]. In particular, single-walled carbon nanotubes (SWCNTs), with their unique one-dimensional tubular structures, offer inherent advantages in ion transport and charge storage, making them ideal electrode materials for high-efficiency supercapacitors [9,10].

Heteroatom doping (N, P, S, B) has been validated as a mature and efficient modification strategy to optimize the electrochemical performance of carbon electrodes [11,12,13]. Existing computational and experimental studies have confirmed that heteroatom doping can modulate the electronic structure of carbon matrices, create abundant defect active sites, and improve surface wettability, thereby enhancing the capacitance and ion diffusion kinetics of carbon-based supercapacitors [14,15,16,17]. As the most widely studied dopants, N and P atoms have unique modification advantages: N doping introduces pyridinic N and quaternary N active sites to enhance the ion adsorption capacity of carbon materials [18,19], while P doping with larger atomic radii induces structural defects and expands interlayer spacing to facilitate ion transport [20,21]. Numerous previous DFT studies have focused on the capacitive performance and ion adsorption behavior of N/P-doped carbon materials.

Nevertheless, current computational studies still have obvious research gaps. Most previous investigations have only qualitatively analyzed the improvement in the electrochemical performance of carbon nanotubes upon N or P doping, lacking a systematic, comparative exploration of the two dopants at identical doping concentrations. More importantly, existing literature rarely focuses on the core link between heteroatom doping and Li^+^ desolvation behavior in organic electrolyte systems. The critical desolvation size of [Li(AN)]^+^ complexes determines the effective pore size utilization and charge storage efficiency of SWCNT electrodes. However, the differential regulatory effects of N and P doping on the Li^+^ desolvation threshold, desolvation energy, and subsequent ion-diffusion barriers remain unclear. In particular, the intrinsic correlation among dopant type, desolvation characteristics, and relative capacitance enhancement has not been quantitatively elucidated, thereby limiting the targeted structural design of doped CNT electrodes for organic supercapacitors.

Li^+^ desolvation is the core rate-limiting step of charge storage in OSCs, directly determining the ion intercalation efficiency and diffusion rate of electrode materials [22]. In acetonitrile-based organic electrolytes, Li^+^ exists stably in the form of solvated [Li(AN)]^+^ complexes, and complete desolvation is a prerequisite for efficient charge storage [23,24]. First-principles DFT calculations can quantitatively reveal the atomic-scale interaction mechanisms between solvated ions and electrode materials [25,26], thereby compensating for the limitations of experimental characterization [27,28]. Based on the above research deficiencies, this work systematically investigates and compares the regulatory effects of equal-concentration N and P doping on the Li^+^ desolvation mechanism, relative capacitance, and ion diffusion kinetics of SWCNTs via DFT calculations. The critical desolvation sizes of [Li(AN)]^+^ in pristine, N-doped, and P-doped SWCNTs are quantitatively determined. Combined with charge-difference density and Mulliken population analyses, the evolution of the electronic structure and the variation in bond strength induced by doping are revealed, thereby fundamentally explaining the performance differences among doped systems. The key novelty of this work is the quantitative clarification, for the first time, of the differential modification mechanisms of N and P doping on Li^+^ desolvation behavior, and the establishment of a clear correlation among dopant type, desolvation characteristics, and supercapacitor capacitive/diffusion performance. This study provides a targeted theoretical basis for the precise design and performance optimization of high-efficiency carbon-based OSC electrodes.

## 2. Calculation Method

In this study, all first-principles calculations and full geometric optimizations were performed using the CASTEP (2019 Version) [29] module based on density functional theory (DFT) [30]. The generalized gradient approximation with the Perdew–Burke–Ernzerhof (GGA-PBE) [31] functional was adopted to describe electron exchange–correlation interactions, and the Grimme van der Waals (vdW) correction was incorporated to accurately characterize the weak intermolecular interactions between solvent molecules, lithium ions, and carbon nanotube substrates, which is essential for simulating ion adsorption and desolvation behavior in organic electrolytes. Ultrasoft pseudopotentials [32] were used to model the valence electron–ion core interactions of C, N, P, Li, and H atoms.

A plane-wave cutoff energy of 400 eV was employed for all computational systems. This cutoff energy was determined by strict convergence tests: increasing the cutoff energy beyond 400 eV caused negligible changes in the total energy and structural parameters of the system (energy fluctuation < 1 × 10^−5^ eV/atom), which balances calculation accuracy and computational efficiency. For the Brillouin zone sampling, a 1 × 1 × 2 [33,34] Monkhorst–Pack k-point grid was selected after systematic convergence verification, suitable for the one-dimensional tubular periodic structure of SWCNTs.

All structural models were constructed based on periodic (5,5) armchair SWCNT supercells, which are typical and widely adopted single-walled carbon nanotube models for electrochemical ion adsorption research. To ensure a fair comparative study, an identical single-atom doping concentration was adopted for both N and P modification systems. Specifically, one carbon atom in the pristine SWCNT supercell was substitutionally replaced by a single N or P heteroatom, forming uniform low-concentration doped structures, which eliminates the interference of doping content differences on electrochemical performance comparison.

For dopant site selection, the heteroatom was substitutionally doped at the stable lattice site on the inner wall of the SWCNT tube, rather than surface adsorption, which conforms to the stable doping configuration of N/P-doped carbon nanotubes reported in previous studies. This internal lattice doping mode can effectively regulate the internal electronic structure and pore microenvironment of SWCNTs, which is the key structural basis for modulating Li^+^ desolvation and diffusion behavior. It should be noted that the nitrogen doping configuration investigated in this work corresponds specifically to graphitic (quaternary) nitrogen, in which a single N atom substitutionally replaces a lattice carbon atom and remains three-fold sp^2^-coordinated within the carbon framework. This configuration differs from pyridinic N and pyrrolic N, which typically occur at edge sites or defect vacancies and involve two-fold or five-membered-ring coordination environments, respectively. The choice of graphitic N in this study ensures a direct and fair comparison with P doping under identical substitutional lattice conditions, while the effects of other N configurations are discussed as future perspectives in Section 3.6. All initial doped models were fully relaxed to obtain the minimum-energy stable structures for subsequent calculation and analysis.

During geometric optimization and energy calculation, strict convergence criteria were set: the maximum interatomic force of 0.03 eV/Å, the internal stress convergence of 5 × 10^−2^ GPa, the total energy convergence of 1 × 10^−5^ eV per atom, and the maximum ionic displacement of 0.01 Å. All computational parameters and model construction strategies follow the standard DFT calculation specifications for carbon-based electrode materials and ion desolvation simulation, ensuring the reliability, accuracy, and reproducibility of the computational results.

In this work, periodic hexagonal supercell models were constructed to simulate various single-walled carbon nanotube (SWCNT) structures. The (5,5) armchair SWCNT was adopted as the typical research model, with heteroatoms doped at the internal central site of the nanotube cavity, as illustrated in Figure 1. In the structural schematic, gray, blue, and green spheres represent carbon, nitrogen, and phosphorus atoms on the pore surface, respectively. The investigated SWCNT systems cover a typical diameter range of 5–10 Å, which fully encompasses the effective pore size scope for lithium-ion adsorption and desolvation investigated in this study, as summarized in Table 1.

## 3. Results and Discussion

### 3.1. Reaction Principle

To quantitatively evaluate the Li^+^ desolvation behavior and intercalation feasibility of solvated ions in pristine and doped SWCNTs, three typical competing reaction pathways during the interaction between [Li(AN)]^+^ complexes and carbon nanotube pores were proposed in this work. This classical reaction classification and interaction energy calculation framework is widely employed in first-principles studies of ion desolvation, adsorption, and intercalation behavior in carbon-based electrode materials for organic electrolyte energy storage systems [35]. The three mutually exclusive reaction modes correspond to complete desolvation, solvent preferential intercalation, and intact solvated ion co-intercalation, which cover all possible interfacial behaviors of solvated lithium ions entering CNT pores, and can effectively judge the critical desolvation threshold of [Li(AN)]^+^ complexes.

During the intercalation process of [Li(AN)]^+^ into SWCNTs or doped SWCNTs (DSWCNTs), three possible competitive reactions occur, as follows: (1) Complete Li^+^ desolvation: solvated complexes decompose, free Li^+^ intercalates into CNT pores, and acetonitrile (AN) solvent molecules escape. (2) Solvent preferential adsorption: AN molecules enter the pores alone while Li^+^ remains outside the tube. (3) Intact complex intercalation: undissociated [Li(AN)]^+^ complexes co-embed into CNT pores. The three reaction pathways are described by Equations (1)–(3), respectively:

(1) Li (AN) + SWCNTs/DSWCNTs → Li (SWCNTs/DSWCNTs) + AN

(2) Li (AN) + SWCNTs/DSWCNTs → AN (SWCNTs/DSWCNTs) + Li

(3) Li (AN) + SWCNTs/DSWCNTs → Li (AN) SWCNTs/DSWCNTs

where Li(AN) represents the acetonitrile-solvated lithium ion complex; SWCNTs and DSWCNTs denote pristine and N/P-doped single-walled carbon nanotubes, respectively; Li(SWCNTs/DSWCNTs), AN(SWCNTs/DSWCNTs), and Li(AN)(SWCNTs/DSWCNTs) correspond to the intercalation configurations of free Li^+^, pure AN solvent, and intact solvated complex within CNT pores, respectively.

To quantitatively compare the thermodynamic feasibility of the above three reactions and determine the critical desolvation pore size, the interaction energies (E) of each reaction pathway were calculated following the standard energy decomposition formula for interfacial adsorption systems [15,35]. The definition of interaction energy strictly follows the principle of product system energy minus reactant system energy. A more negative interaction energy indicates a more thermodynamically favorable reaction process and a more stable final adsorption configuration. The corresponding calculation formulas are defined as Equations (4)–(8):

(4) E_int1_ = E_Li(DSWCNTs)_ + E_AN_ − E_Li(AN)_ − E_DSWCNTs_

(5) E_int2_ = E_AN(DSWCNTs)_ + E_Li_ − E_Li(AN)_ − E_DSWCNTs_

(6) E_int3_ = E_Li (AN) DSWCNTs_ − E_Li(AN)_ − E_DSWCNTs_

(7) E_int4_ = E_Li(SWCNTs)_ + E_AN_ − E_Li(AN)_ − E_SWCNTs_

(8) E_int5_ = E_Li (AN) SWCNTs_ − E_Li(AN)_ − E_SWCNTs_

The physical definitions of all energy terms involved in the above formulas are clarified as follows: E_Li(AN)_ represents the total energy of acetonitrile-solvated lithium ion complexes; E_DSWCNTs_ and E_SWCNTs_ correspond to the static total energies of doped and pristine single-walled carbon nanotube substrates, respectively; E_Li(DSWCNTs)_ and E_Li(SWCNTs)_ refer to the total energies of stable composite configurations after free Li^+^ intercalation into doped and pristine SWCNTs, respectively; and E_AN_ stands for the system energy of acetonitrile molecules adsorbed within doped SWCNTs. In addition, E_AN(DSWCNTs)_ and E_Li(AN) SWCNTs_ are the total energies of composite systems formed by intact solvated [Li(AN)]^+^ complexes embedded in pristine and doped SWCNTs, respectively. E_Li_ and E_Li(AN)DSWCNTs_ individually denote the intrinsic energies of isolated acetonitrile solvent molecules and free lithium ions. In this theoretical system, a more negative interaction energy value corresponds to a more thermodynamically spontaneous reaction process and a more stable final adsorption/intercalation configuration.

### 3.2. Desolvation of Li^+^ Complexes by Doped Single-Walled Carbon Nanotubes

#### 3.2.1. Desolvation of Li^+^ Complexes by Nitrogen-Doped Single-Walled Carbon Nanotubes

Figure 2 presents the energy profiles for the desolvation reaction of [Li(AN)]^+^ on pristine single-walled carbon nanotubes (SWCNTs) and nitrogen-doped SWCNT (N-doped SWCNTs) models. To identify the critical pore size that enables the desolvation of [Li(AN)]^+^, we systematically calculated the reaction energies of Li^+^, free AN molecules, and [Li(AN)]^+^ complexes confined in N-doped SWCNTs, as well as the reaction energies of Li^+^ and [Li(AN)]^+^ within pristine SWCNTs. These five energy parameters were sequentially defined as E_int1_, E_int2_, E_int3_, E_int4_, and E_int5_ in this work.

For N-doped SWCNTs with a tube diameter below 6.26 Å, the negative value of E_int2_ verifies the stable existence of individual acetonitrile (AN) molecules within the nanotube cavity. A critical desolvation threshold of 5.91 Å is identified for [Li(AN)]^+^ complexes in N-doped SWCNTs, as nearly equivalent energy values of E_int1_ (−2.83 eV) and E_int3_ (−2.88 eV) are obtained at this pore size. For nanotube diameters smaller than 5.91 Å, the thermodynamic relationship E_int1_ < E_int3_ < 0 is satisfied, demonstrating that free Li^+^ possess higher structural stability inside the tube pores compared with solvated [Li(AN)]^+^ complexes. Accordingly, full desolvation of encapsulated [Li(AN)]^+^ complexes can be spontaneously achieved in N-doped SWCNTs with pore diameters below 5.91 Å.

For pristine SWCNTs, the pore size of 5.91 Å serves as the critical desolvation threshold for [Li(AN)]^+^ solvated complexes, where the calculated E_int4_ (−2.98 eV) and E_int5_ (−3.06 eV) values are highly approximate. At pore sizes narrower than 5.91 Å, the inequality of E_int4_ < E_int5_ < 0 holds thermodynamically, which demonstrates that free Li^+^ exhibits superior stability within the tubular pores relative to intact [Li(AN)]^+^ clusters. On this basis, pristine SWCNTs with a diameter smaller than 5.91 Å are capable of realizing thorough desolvation of internally confined [Li(AN)]^+^ complexes.

#### 3.2.2. Desolvation of Li^+^ Complexes by Phosphorus-Doped Single-Walled Carbon Nanotubes

The energy profiles describing the desolvation behaviors of [Li(AN)]^+^ complexes within pristine and phosphorus-doped single-walled carbon nanotubes (P-doped SWCNTs) are plotted in Figure 3. In this work, five key reaction energy parameters (E_int1_, E_int2_, E_int3_, E_int4_, and E_int5_) were systematically computed to pinpoint the threshold pore size enabling complete [Li(AN)]^+^ desolvation. Specifically, the calculation covered the reaction energies of Li^+^, free AN solvent molecules, and solvated [Li(AN)]^+^ complexes in P-doped SWCNTs, as well as the energetic responses of Li^+^ and [Li(AN)]^+^ in undoped pristine SWCNTs.

Calculation results reveal that E_int2_ presents negative values for P-doped SWCNTs with diameters under 6.26 Å, proving that free AN solvent molecules can stably reside within the tubular structure. The pore size of 6.11 Å is determined as the critical desolvation boundary for [Li(AN)]^+^ complexes in P-modified SWCNTs, owing to the comparable energetic magnitudes between Eint1 (−4.29 eV) and Eint3 (−4.22 eV) at this dimension. For P-doped nanotubes with apertures smaller than 6.11 Å, the thermodynamic trend E_int1_ < E_int3_ < 0 is consistently observed, suggesting that free lithium ions achieve more stable adsorption within pore channels than solvated [Li(AN)]^+^ clusters. As a result, full dissociation and desolvation of trapped [Li(AN)]^+^ complexes can be fully realized in P-doped SWCNTs with pore diameters below 6.11 Å.

### 3.3. Analysis of the Effect of Doped Carbon Nanotubes on the Desolvation Size of Li^+^

To analyze the critical desolvation size of Li^+^ complexes, Figure 4 presents the relationship between doped carbon nanotubes and the desolvation size of Li^+^ complexes. In the acetonitrile-based solvent system, the fully desolvated size of Li^+^ complexes is 5.91 Å for both pristine SWCNTs and N-doped SWCNTs. In comparison, P-doped SWCNTs exhibit an expanded critical desolvation size of 6.11 Å. This doping-induced increase in the critical desolvation threshold serves as the core physical origin of improved Li^+^ storage capacity and capacitive performance of PCNTs.

The enlarged complete desolvation size of P-doped SWCNTs significantly broadens the valid pore size range for effective Li^+^ storage. For pristine and N-doped SWCNTs, only pores smaller than 5.91 Å can realize thorough desolvation and accommodate free Li^+^ for charge storage. By contrast, PCNTs can achieve complete [Li(AN)]^+^ desolvation within an extended pore range below 6.11 Å. Such expansion allows more tubular pore structures to participate in effective ion adsorption and charge accumulation, greatly increasing the number of available active sites for Li^+^ storage. Furthermore, the weakened P-C covalent bonding and reduced electron localization in PCNTs mitigate structural steric hindrance and electronic restriction toward Li^+^, enabling more stable ion embedding and accommodation inside the tube cavity. Benefiting from the expanded valid desolvation window and optimized ion affinity, P-doped SWCNTs possess higher effective Li^+^ loading and storage efficiency, which macroscopically contributes to the prominent enhancement of supercapacitor relative capacitance.

### 3.4. Relative Capacitance Analysis of Carbon Nanotubes Doped with Li^+^ Intercalation

This section investigates the intercalation of Li^+^ (lithium ions) into carbon nanotubes (CNTs) with different diameters and functionalized carbon nanotubes, under the condition that Li^+^ does not bind to solvent molecules. Figure 5, Figure 6 and Figure 7 show the optimized structures of Li^+^ intercalated into carbon nanotubes with different pore sizes and doped carbon nanotubes, respectively.

Structural optimization results from Figure 6 and Figure 7 clearly illustrate the geometric configuration of Li^+^ intercalation within N- and P-doped SWCNTs. For nanotubes with small pore diameters of 5.42 Å and 5.65 Å, desolvated Li^+^ are stably confined at the geometric center of the tubular cavity, presenting a highly symmetric adsorption state. With the gradual enlargement of pore size, the central confinement effect weakens, causing the embedded Li^+^ to spontaneously shift toward the inner tube wall. Accordingly, the average distance between Li^+^ and the carbon framework continuously decreases, which alters the interfacial contact state and charge interaction strength between ions and the electrode substrate.

To quantitatively evaluate the capacitive performance of different SWCNT configurations, the classical electric double-layer capacitor (EDLC) theoretical formula [36,37] was adopted for performance correlation analysis:

(9) $$C_{EDLC}=\frac{\varepsilon A}{Rln\frac{R}{R-d1}}$$

In this equation, C represents the theoretical double-layer capacitance, ε is the local dielectric constant of the organic electrolyte, A refers to the effective specific surface area of the electrode, *d* is the ionic radius of Li^+^, and R corresponds to the tubular radius of pristine and heteroatom-doped SWCNTs. Considering that the actual capacitive contribution originates from desolvated lithium ions rather than solvent-coordinated ion clusters, this study adopts the Li^+^–carbon atom distance (*d*) as a core structural parameter to define the relative capacitance, which enables intuitive and horizontal comparison of charge storage capability among different SWCNT systems. All distance data were statistically processed based on the minimum spacing between Li^+^ and the inner tube wall to ensure data accuracy and consistency. As demonstrated in Figure 8a,b, the variation of relative capacitance is strongly dependent on the nanotube pore size, wherein the spatial location and interfacial bonding state of intercalated Li^+^ fundamentally determine the charge accumulation efficiency and double-layer capacitive behavior of carbon nanotube electrodes.

From the relative capacitance values in Figure 8b, it can be observed that under the same pore size, when Li^+^ is intercalated into phosphorus-doped carbon nanotubes (P-CNTs), the resulting relative capacitance is significantly higher than that of the intrinsic system. However, when Li^+^ is intercalated into nitrogen-doped carbon nanotubes (N-CNTs), it cannot be guaranteed that the relative capacitance is higher than that of the intrinsic system under all pore sizes.

Consistent with the above computational measurements, both pristine carbon nanotubes and nitrogen-modified carbon nanotubes share an identical critical pore dimension of 5.91 Å for the full desolvation of Li^+^ solvated complexes. In comparison, it is 6.11 Å for phosphorus-doped carbon nanotubes (P-CNTs). For the doped carbon nanotube systems with pore sizes smaller than the desolvation size, their relative capacitance values are all higher than those of the intrinsic system, and the increase in relative capacitance is particularly significant for the P-CNT system.

As shown in Table 2, when Li^+^ undergoes desolvation in P-CNTs, the resulting relative capacitance exceeds 1.2 times that of the intrinsic system, reaching a maximum of 1.9 times. In contrast, when Li^+^ undergoes desolvation in N-CNTs, the relative capacitance exceeds 1.1 times that of the intrinsic system, reaching a maximum of 1.3 times. These analytical results demonstrate that the expansion of the lithium solvated complex desolvation window effectively improves the charge storage capacity of doped carbon nanotube electrodes. Broadened desolvation dimensions correspond to higher relative capacitance outputs of the modified carbon nanotube systems, and the stripping of solvent ligands from solvated Li+ complexes significantly enhances the capacitive performance of electrode materials.

### 3.5. Influence of N/P Doping on the Diffusion Energy Barrier of [Li(AN)]^+^ in Carbon Nanotubes

To investigate the diffusion performance of [Li(AN)]^+^ in doped carbon nanotubes (CNTs) and pristine carbon nanotubes (CNTs), their diffusion energy barriers were calculated sequentially. As shown in Figure 9a, when [Li(AN)]^+^ diffuses along the A-to-B path in N-doped carbon nanotubes, the diffusion energy barrier is 1.12 eV; when [Li(AN)]^+^ diffuses along the C-to-D path in pristine CNTs, the diffusion energy barrier is 1.13 eV. There is no significant change in the diffusion energy barrier of [Li(AN)]^+^ when comparing N-doped CNTs with pristine CNTs. After doping CNTs with N atoms, the diffusion energy barrier of [Li(AN)]^+^ remains unchanged, indicating that the presence of N atoms does not hinder the diffusion of [Li(AN)]^+^.

As shown in Figure 9b, when [Li(AN)]^+^ diffuses along the A-to-B path in P-doped carbon nanotubes, the diffusion energy barrier is 0.99 eV; compared with that in pristine CNTs, the diffusion energy barrier of [Li(AN)]^+^ decreases significantly. Therefore, [Li(AN)]^+^ diffuses more easily in P-doped carbon nanotubes than in pristine CNTs. After doping CNTs with P atoms, the diffusion energy barrier of [Li(AN)] ^+^ is reduced, which indicates that the presence of P atoms also does not hinder the diffusion of [Li(AN)] ^+^.

### 3.6. Charge Difference Density Analysis

To further investigate the changes in charge distribution between doped atoms and carbon atoms in the doped system after desolvation, the charge difference density of the doped system at the desolvation size was calculated. As shown in Figure 10, these are the charge-difference density diagrams of the doped system before and after desolvation (the XY-plane and XZ-plane are projected with the doped atom as the center), where the red and blue regions represent electron accumulation and depletion, respectively.

As shown in Figure 10, the region near Li^+^ after desolvation appears blue, indicating that Li^+^ loses electrons. Compared with the intrinsic single-walled carbon nanotube system, the red regions shrink and fade in color after doping with N and P, while the blue regions tend to expand. The faded color of the surrounding areas and the increased blue area caused by doping indicate that electron accumulation around the atoms is reduced and electron localization is weakened, further demonstrating that the strengths of the N-C and P-C bonds decrease. Among these, the P-C bond exhibits a larger blue area than the N-C bond, with weaker electron locality and a more significant reduction in bond strength.

To further reveal the intrinsic electronic mechanism governing Li^+^ desolvation, capacitive enhancement, and ion diffusion kinetics, charge difference density analysis was performed to characterize the electronic redistribution after N and P doping. As shown in Figure 10, desolvated Li^+^ exhibits obvious electron depletion behavior, confirming positive ionic adsorption characteristics. Compared with pristine SWCNTs, both N-doping and P-doping induce contracted electron accumulation regions and expanded electron depletion regions, indicating that heteroatom doping effectively breaks the original highly localized electron distribution of pure carbon frameworks and reduces local electron density. Such electronic delocalization weakens the covalent strength of N-C and P-C bonds, and the P-C bonding presents a more significant weakening effect than N-C bonding.

This doping-induced electronic structural change is the essential origin of improved desolvation behavior, capacitive performance, and diffusion kinetics. First, the reduced electron localization and weakened framework bonding decrease the surface electronic repulsion between the carbon tube wall and polar [Li(AN)]^+^ complexes, which alleviates the steric and electronic restriction on solvated ions inside CNT pores. This allows larger solvated ion clusters to achieve stable desolvation, thereby increasing the critical desolvation size of PCNTs from 5.91 Å to 6.11 Å. Second, the delocalized electronic structure provides more continuous and uniform active sites for Li^+^ adsorption, stabilizes free lithium ions after desolvation, and improves the effective ion storage density within pores, which directly promotes the relative capacitance of doped CNTs, especially for P-doped systems with stronger electronic regulation ability. Third, weakened P-C covalent interaction softens the rigidity of the carbon lattice and reduces the energy fluctuation during ion migration, which significantly lowers the [Li(AN)]^+^ diffusion barrier to 0.99 eV and accelerates ion transport. In contrast, N-C bonds maintain relatively stronger covalent characteristics, resulting in limited electronic modulation effects, negligible changes in desolvation size, and only slight optimization of diffusion and capacitive performance. Therefore, the distinct electronic redistribution and bond strength differences between N-doping and P-doping fundamentally explain why P-doped SWCNTs possess superior desolvation tolerance, higher charge storage capacity, and faster ion diffusion kinetics, which is highly consistent with the thermodynamic and kinetic results obtained in this study.

It is important to acknowledge a limitation of the current N-doping model. In real carbonaceous materials, nitrogen dopants can exist in multiple configurations, including pyridinic N, pyrrolic N, and graphitic (quaternary) N. The present work focuses exclusively on graphitic N, in which the N atom substitutionally occupies a lattice carbon site and maintains three-fold sp^2^ coordination. Pyridinic N, typically located at edge sites or vacancies with two-fold coordination, possesses a lone electron pair and exhibits stronger Lewis basicity, which may enhance Li^+^ adsorption affinity but could also increase local steric hindrance for solvated ion transport. Pyrrolic N, incorporated into five-membered rings, contributes two p electrons to the π system and may alter the electron density distribution differently from graphitic N. Consequently, the regulatory effects of pyridinic and pyrrolic N on the critical desolvation size, relative capacitance, and diffusion barrier of [Li(AN)]^+^ may differ from those of graphitic N reported here. A systematic comparative study encompassing all three N configurations, ideally combined with experimental XPS characterization to quantify their relative proportions, represents an important direction for future research and would further strengthen the practical guidance of theoretical calculations for experimental electrode design.

## 4. Conclusions

In this work, first-principles calculations were performed to systematically investigate the desolvation behavior and relative capacitance of Li^+^ complexes in pristine and heteroatom-doped carbon nanotubes (CNTs), with acetonitrile (AN) as the solvent. The diffusion characteristics of [Li(AN)]^+^ complexes in N-doped and P-doped CNTs were comprehensively compared with those in pristine CNTs. Differential charge density analysis was employed to characterize the local electronic redistribution induced by heteroatom doping. Meanwhile, the bonding strength between carbon and dopant atoms was evaluated, and the covalent nature of dopant-related chemical bonds was further clarified via Mulliken population analysis. The major findings of this study are summarized as follows:

- First, the fully desolvated diameters of Li^+^ complexes were determined to be 5.91 Å for pristine CNTs, 5.91 Å for N-doped CNTs, and 6.11 Å for P-doped CNTs. Comparative analysis of relative capacitance demonstrates that heteroatom doping effectively improves the capacitive performance of CNTs. Notably, P-doped CNTs exhibit the most prominent enhancement in relative capacitance during the Li^+^ desolvation process, achieving a maximum 1.9-fold improvement compared with pristine CNTs.
- Second, P-doped CNTs exhibit an obvious reduction in the diffusion energy barrier of [Li (AN)]^+^ complexes compared with pristine CNTs. By contrast, the diffusion barrier of N-doped CNTs is almost identical to that of pristine CNTs, and the 0.01 eV difference is within the DFT calculation tolerance, meaning N doping shows no notable modulation on ion migration. Among all configurations, P-doped CNTs deliver the lowest diffusion energy barrier, which endows the [Li (AN)]^+^ complexes with the fastest diffusion kinetics.
- Third, post-desolvation differential charge density calculations reveal that heteroatom doping reduces the local electron density around adjacent atoms and weakens the bonding strength between carbon and dopant atoms. Furthermore, population analysis verifies that the chemical bonds between P and C atoms exhibit the weakest covalency among all tested configurations. It should be emphasized that the conclusions regarding N doping in this work are specifically applicable to graphitic (substitutional) nitrogen; the effects of pyridinic and pyrrolic N configurations, which are common in real carbon materials, remain to be systematically investigated in future studies.

This study reveals the regulatory mechanism of doped carbon nanotubes on lithium-ion desolvation behavior and clarifies the intrinsic mechanism for improving the electrochemical performance of organic system supercapacitors. It provides an important theoretical basis and technical support for the development and application of high-performance organic supercapacitors and auxiliary energy storage devices for electric vehicles.

## Figures and Tables

**Figure 1:**
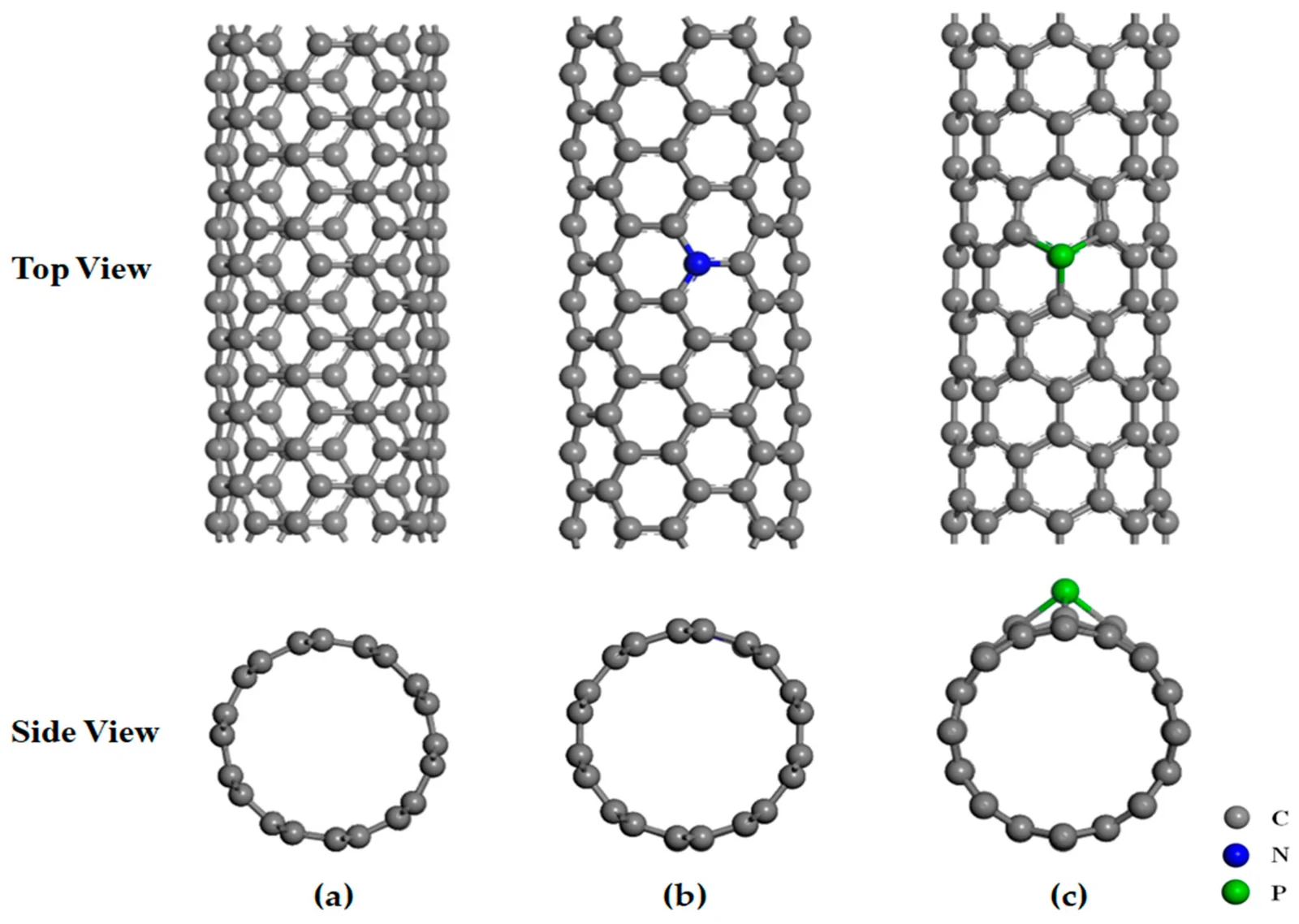
Stable structure of doped single-walled carbon nanotubes. (**a**) Stable structure of the pristine (5,5) single-walled carbon nanotube. (**b**) Stable structure of N(5,5)-doped single-walled carbon nanotube. (**c**) Stable structure of P(5,5)-doped single-walled carbon nanotubes.

**Figure 2:**
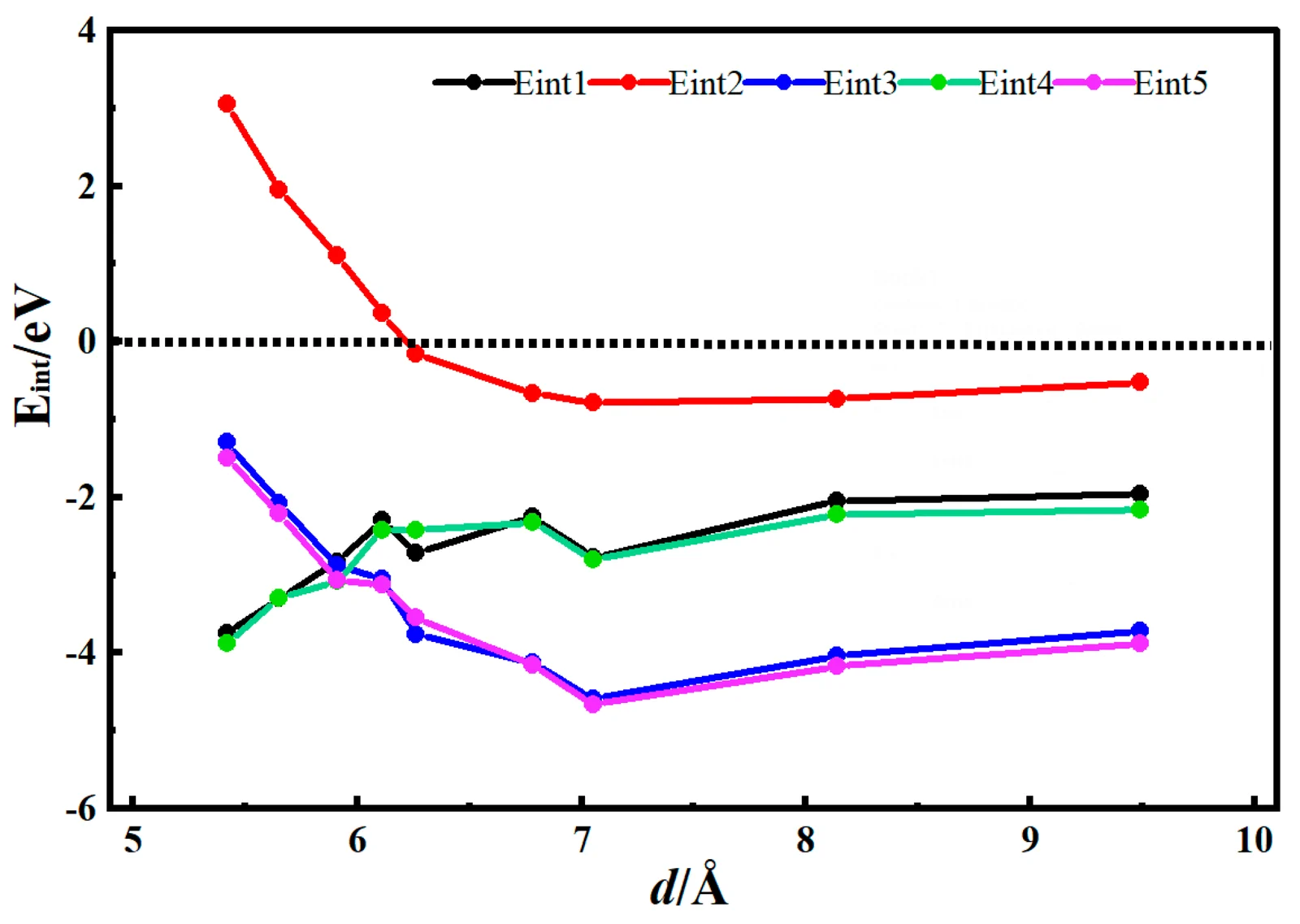
Reaction energy profiles of Li^+^, AN, and [Li(AN)]^+^ in nitrogen-doped carbon nanotubes, as well as Li^+^ and [Li(AN)]^+^ in pristine carbon nanotubes.

**Figure 3:**
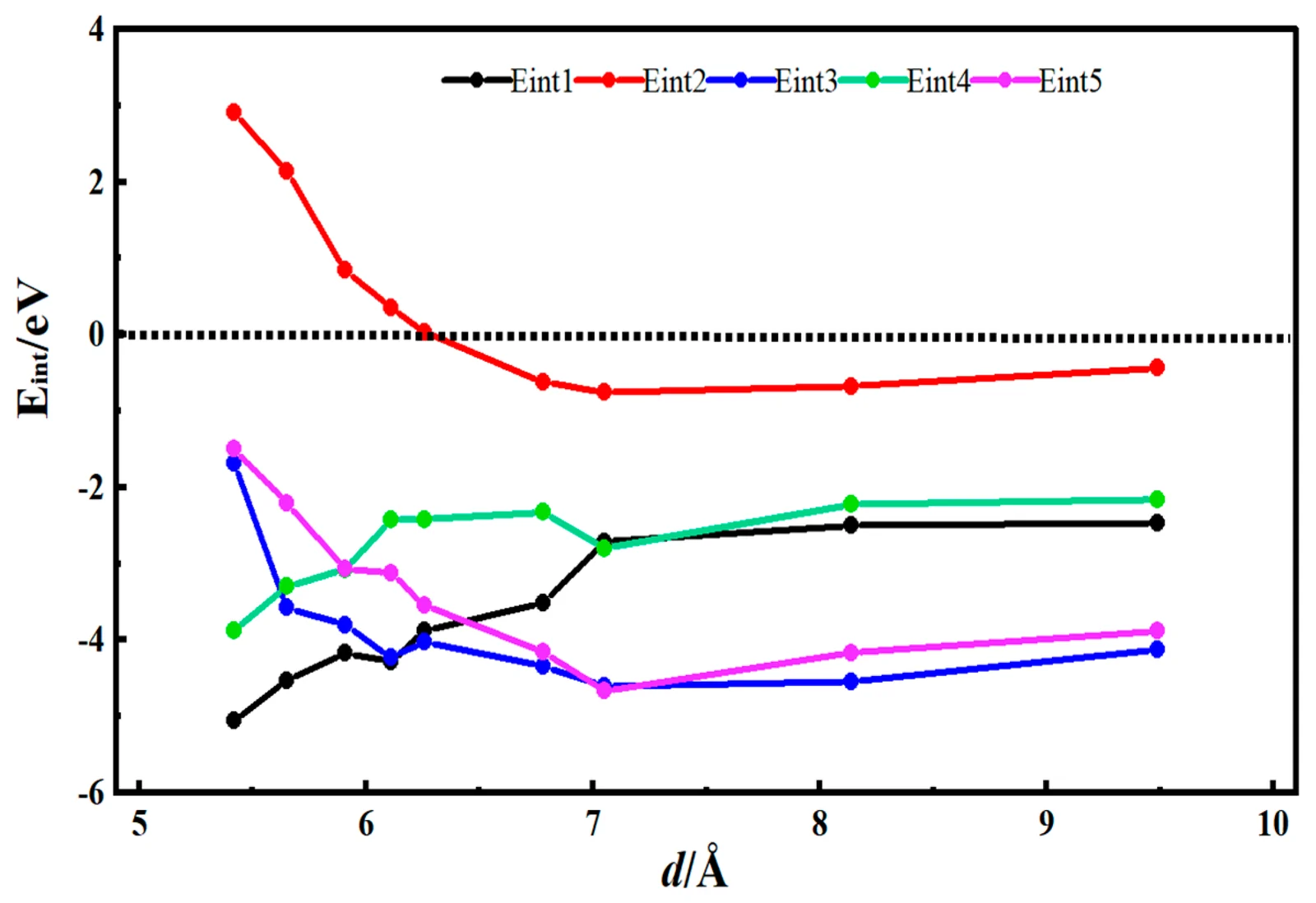
Reaction energy profiles of Li^+^, AN, and [Li(AN)]^+^ in phosphorus-doped carbon nanotubes, as well as Li^+^ and [Li(AN)]^+^ in pristine carbon nanotubes.

**Figure 4:**
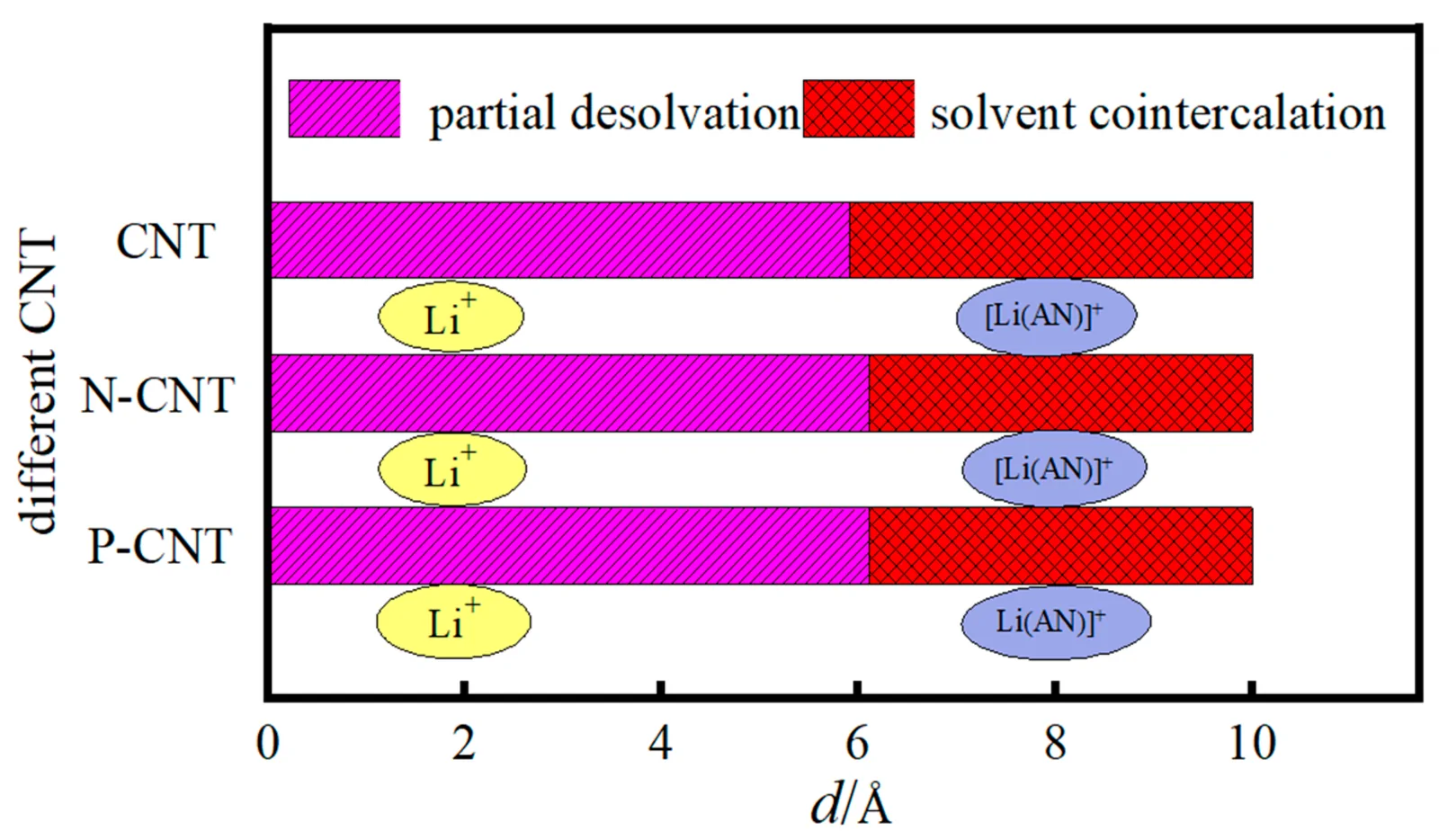
Correlation between the desolvation size of Li^+^ complexes and functionalized-doped carbon nanotubes with different functional groups.

**Figure 5:**
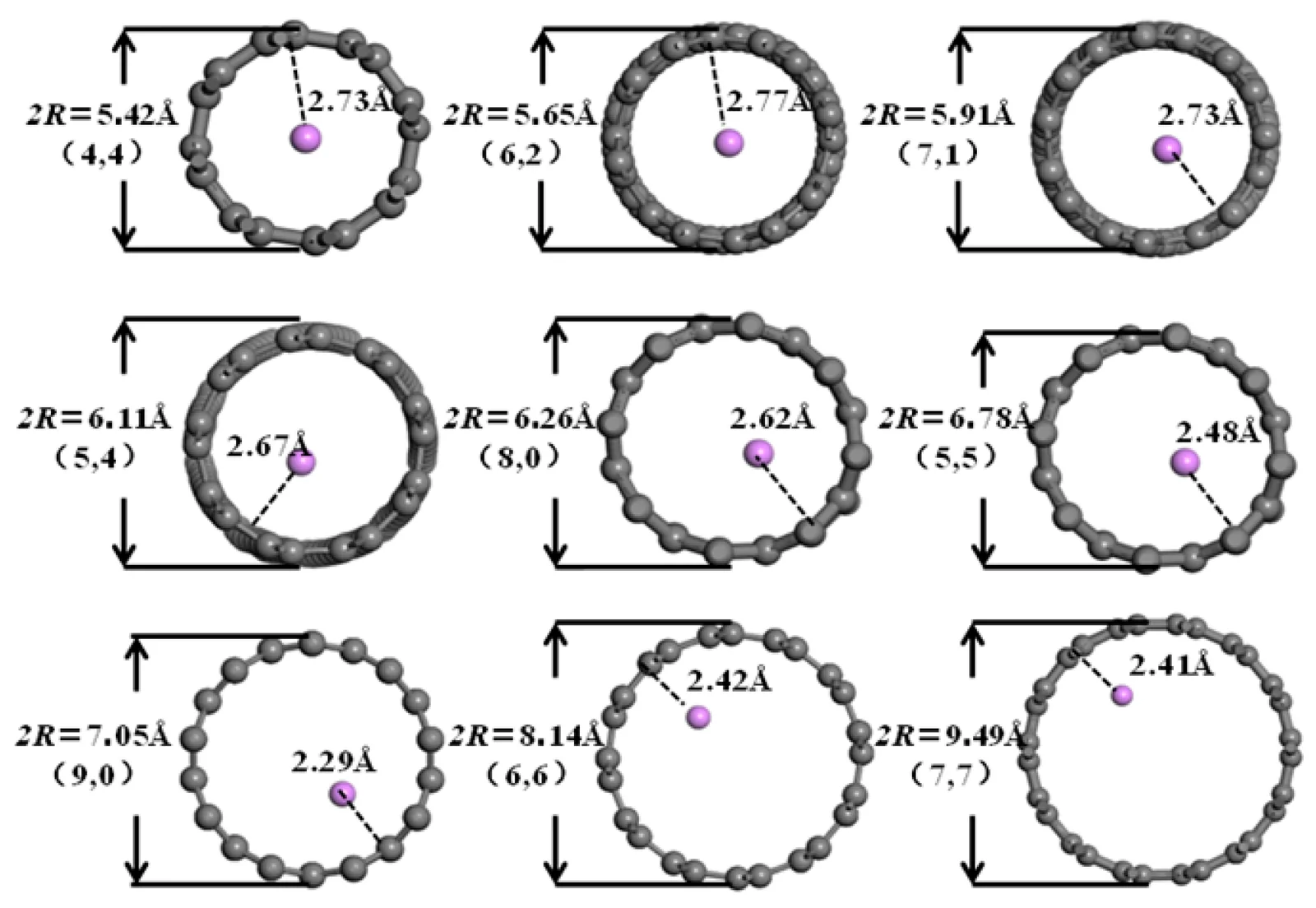
The optimized structures of CNT-Li^+^ [35].

**Figure 6:**
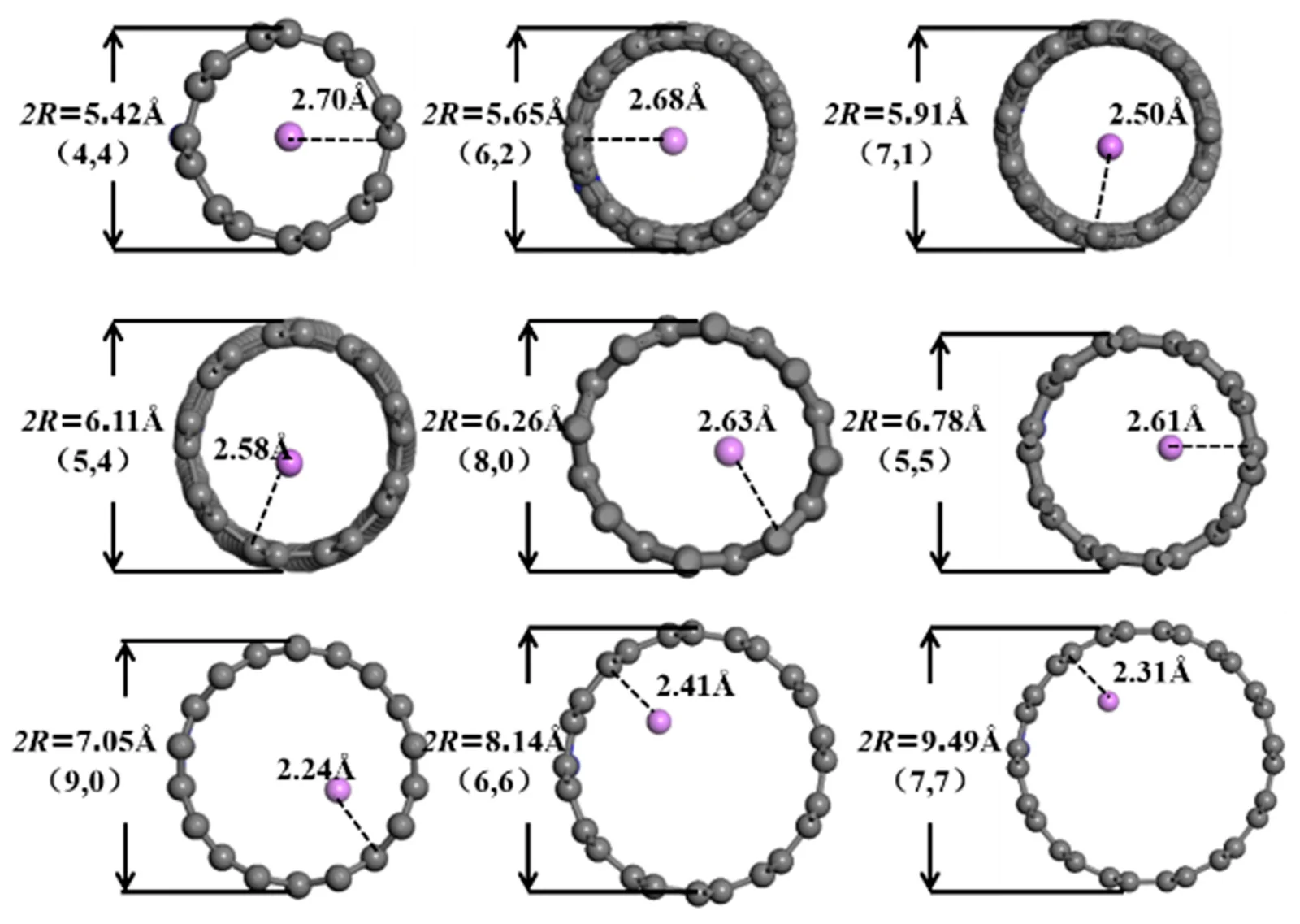
The optimized structures of N-CNT- Li^+^.

**Figure 7:**
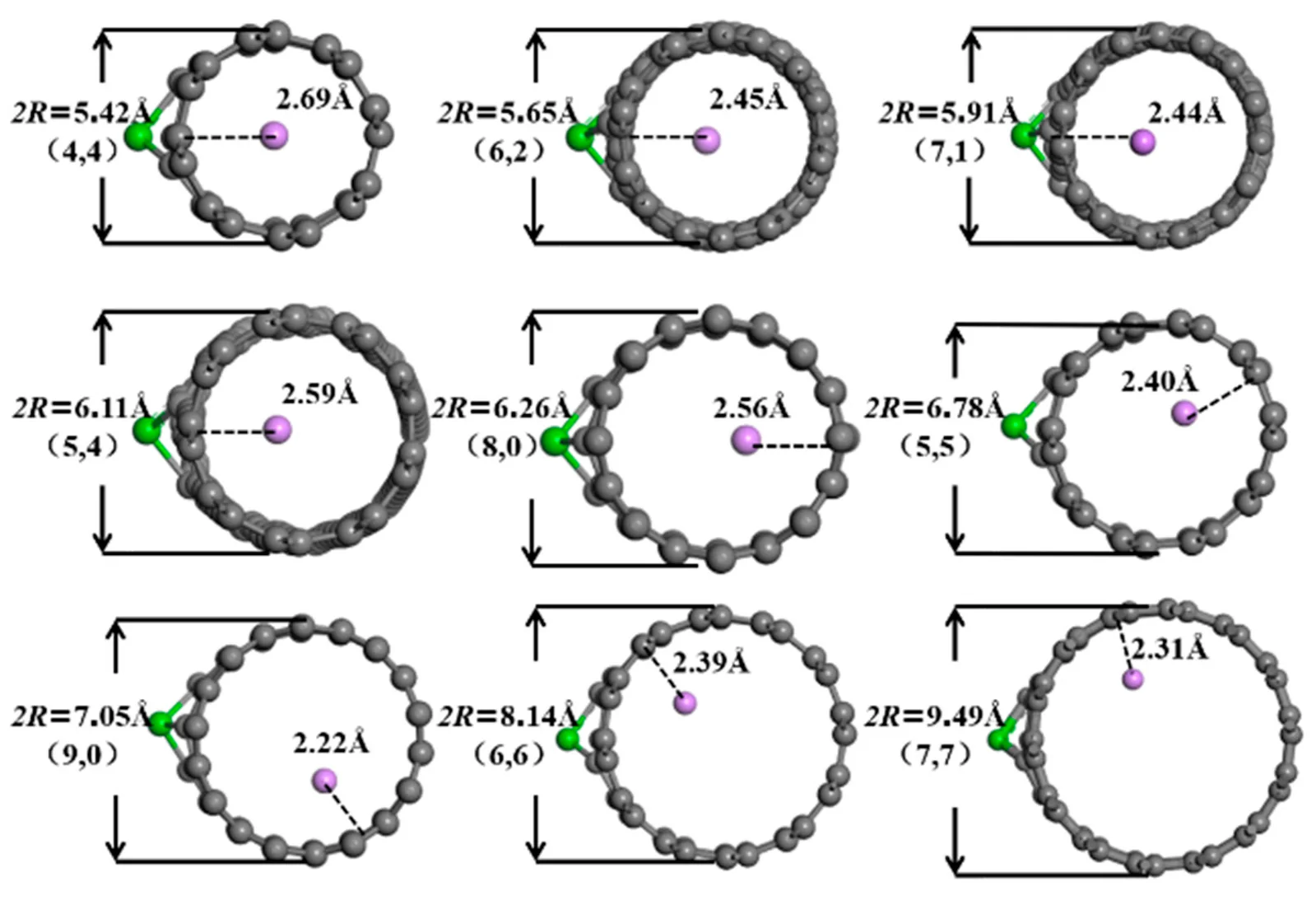
The optimized structures of P-CNT- Li^+^.

**Figure 8:**
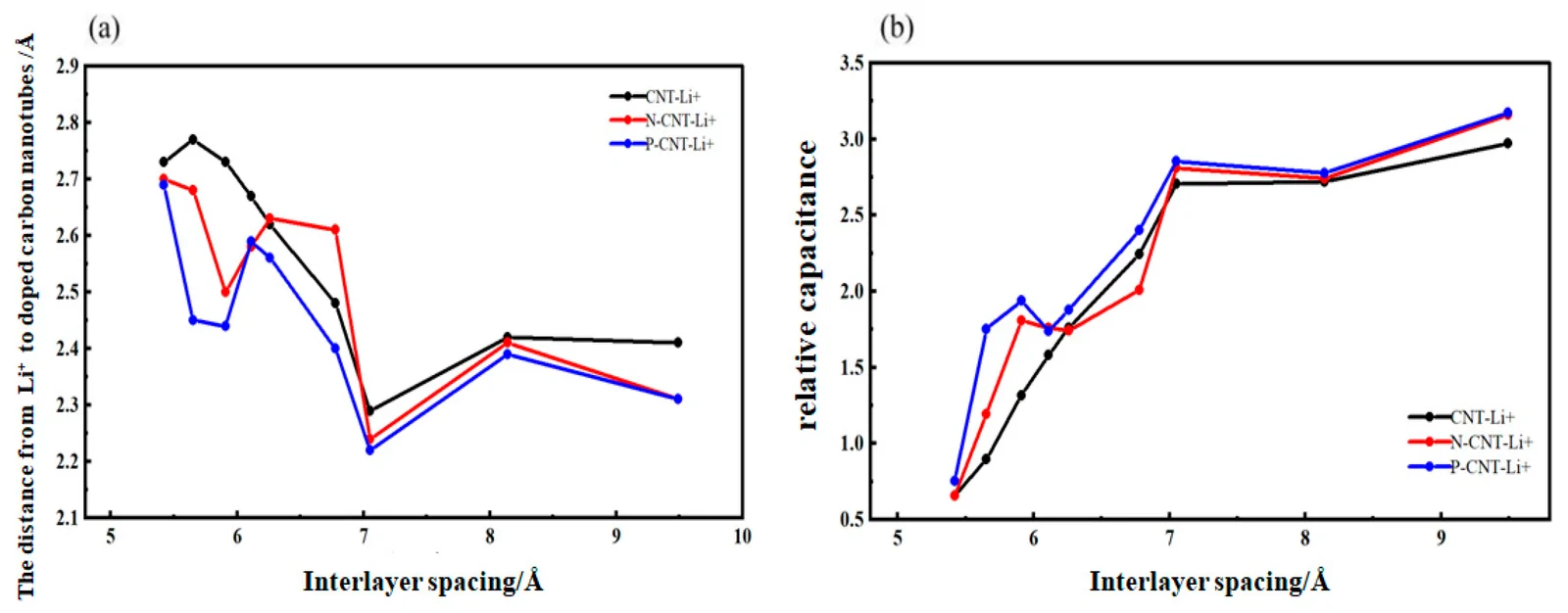
*d*_Li_^+^_-C_ and relative capacitance of Li^+^ embedded into CNT and doped CNT. (**a**) *d*_Li_^+^_-C_ for Li^+^ intercalation into carbon nanotubes and doped carbon nanotubes. (**b**) relative capacitance values.

**Figure 9:**
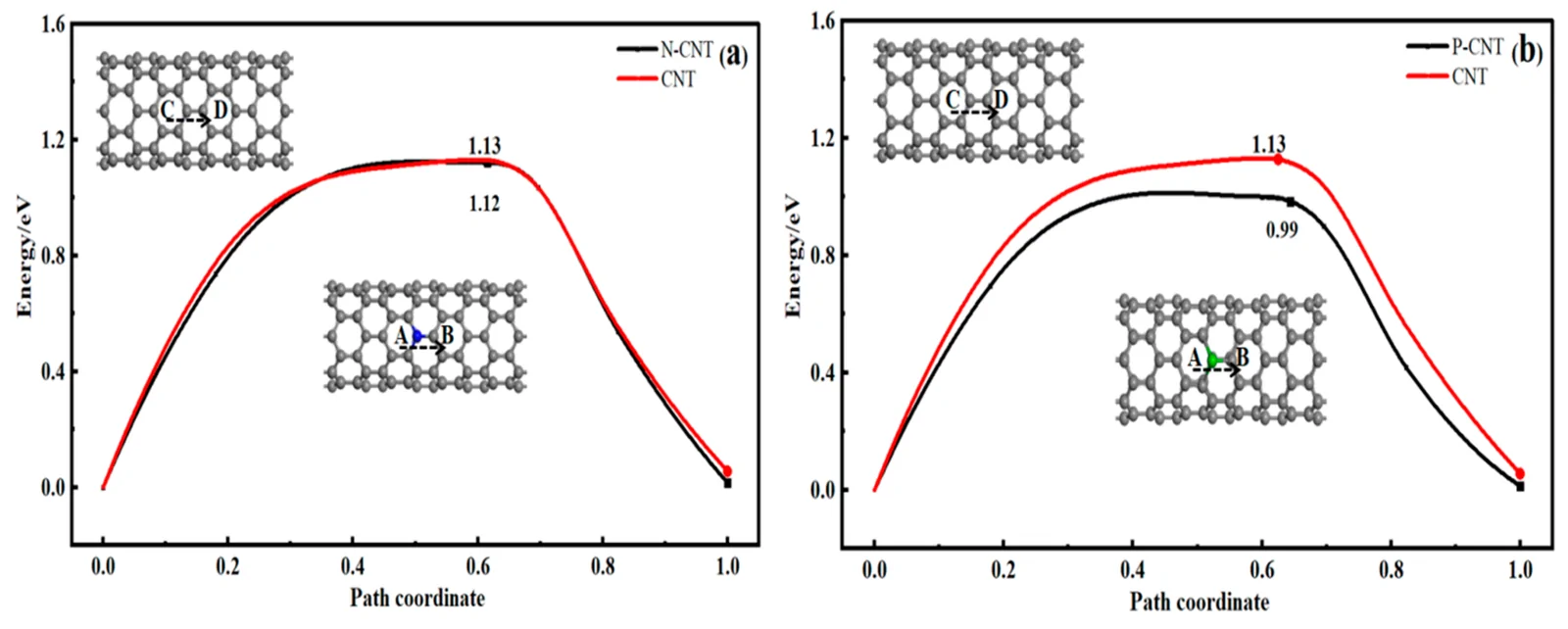
Diffusion energy barrier of [Li(AN)]^+^ in doped CNT and CNT. (**a**) N-doped CNT and CNT. (**b**) P-doped CNT and CNT.

**Figure 10:**
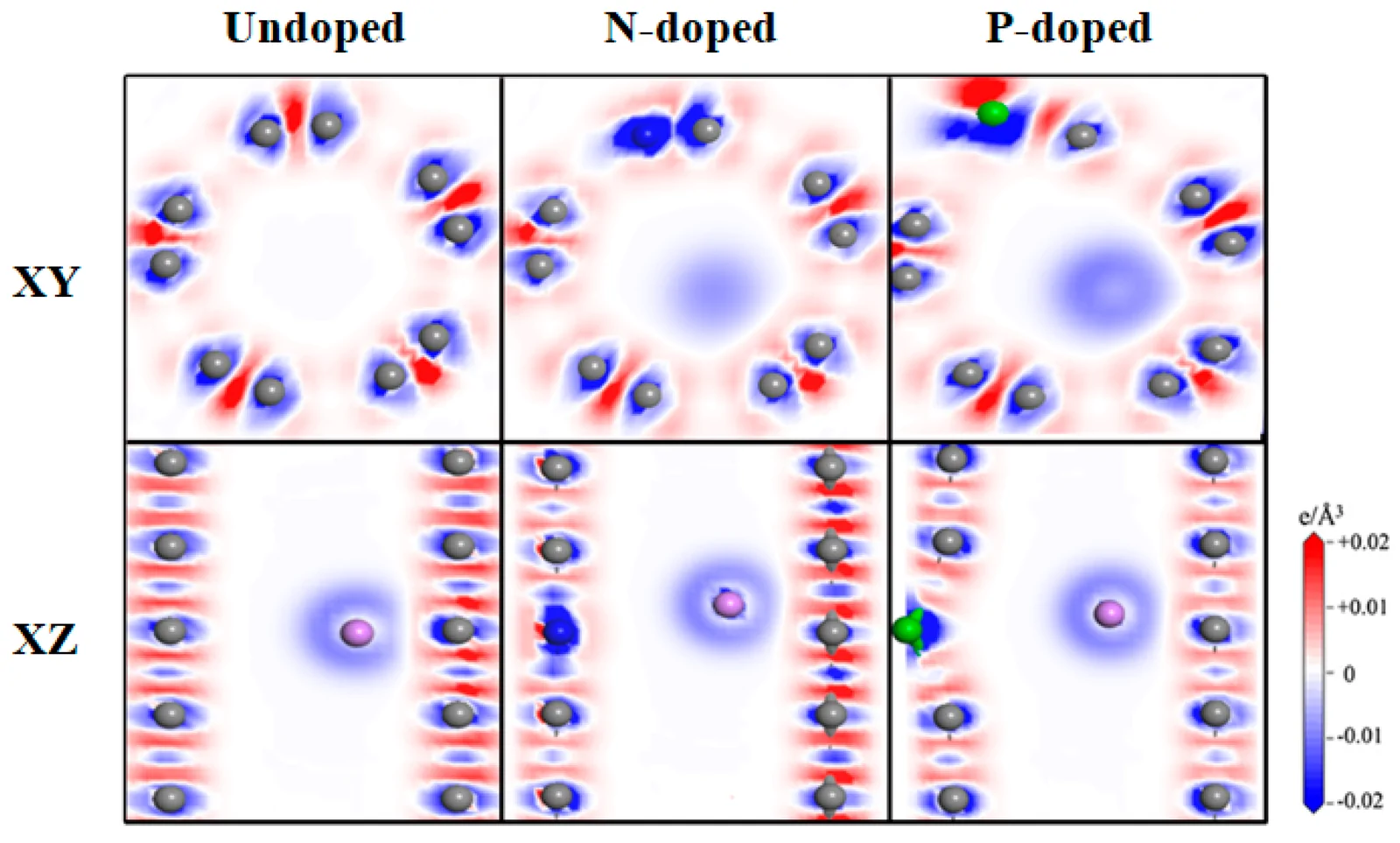
Charge differential density distribution of pure carbon nanotubes and doped carbon nanotubes under desolvation critical size.

**Table 1:** Structural morphologies and diameters of single-walled carbon nanotubes [35].

Structure Form	Armchair	Chiral	Chiral	Chiral	Zigzag	Armchair	Zigzag	Armchair	Armchair
Chiral index	4:4	6:2	7:1	5:4	8:0	5:5	9:0	6:6	7:7
Diameter/Å	5.42	5.65	5.91	6.11	6.26	6.78	7.05	8.14	9.49

**Table 2:** The relative capacitance parameters of CNT- Li^+^ and doped CNT- Li^+^.

Structure	Undoped	N-Doped	P-Doped
CNT-5.42@ Li^+^	0.66	0.72	0.75
CNT-5.65@ Li^+^	0.89	1.19	1.75
CNT-5.91@ Li^+^	1.31	1.81	1.94
CNT-6.11@ Li^+^	1.58	1.76	1.74
CNT-6.26@ Li^+^	1.76	1.74	1.88
CNT-6.78@ Li^+^	2.24	2.01	2.39
CNT-7.05@ Li^+^	2.70	2.81	2.85
CNT-8.14@ Li^+^	2.72	2.74	2.78
CNT-9.49@ Li^+^	2.97	3.16	3.17

## Data Availability

The original contributions presented in this study are included in the article. Further inquiries can be directed to the corresponding author.
